# Interleukin-6 increases matrix metalloproteinase-14 (MMP-14) levels via down-regulation of p53 to drive cancer progression

**DOI:** 10.18632/oncotarget.11243

**Published:** 2016-08-12

**Authors:** Jillian M. Cathcart, Anna Banach, Alice Liu, Jun Chen, Michael Goligorsky, Jian Cao

**Affiliations:** ^1^ Division of Cancer Prevention, Department of Medicine, Stony Brook University, Stony Brook, NY, USA; ^2^ Molecular and Cellular Pharmacology Program, Department of Pharmacology, Stony Brook University, Stony Brook, NY, USA; ^3^ Department of Medicine, Renal Research Institute, New York Medical College, Valhalla, NY, USA

**Keywords:** MMP-14, IL-6, metastasis, p53

## Abstract

Matrix metalloproteinases (MMPs) play critical roles in cancer invasion and metastasis by digesting basement membrane and extracellular matrix (ECM). Much attention has focused on the enzymatic activities of MMPs; however, the regulatory mechanism of MMP expression remains elusive. By employing bioinformatics analysis, we identified a potential p53 response element within the MMP-14 promoter. Experimentally, we found that p53 can repress MMP-14 promoter activity, whereas deletion of this p53 response element abrogated this effect. Furthermore, we found that p53 expression decreases MMP-14 mRNA and protein levels and attenuates MMP-14-mediated cellular functions. Additional promoter analysis and chromatin immunoprecipitation studies identified a mechanism of regulation of MMP-14 expression by which p53 and transcription factor Sp1 competitively bind to the promoter. As the correlation between inflammation and cancer aggressiveness is well described, we next sought to evaluate if inflammatory cytokines could differentially affect p53 and MMP-14 levels. We demonstrate that interleukin-6 (IL-6) down-regulates p53 protein levels and thus results in a concomitant increase in MMP-14 expression, leading to enhanced cancer cell invasion and metastasis. Our data collectively indicate a novel mechanism of regulation of MMP-14 by a cascade of IL-6 and p53, demonstrating that the tumor microenvironment directly stimulates molecular changes in cancer cells to drive an invasive phenotype.

## INTRODUCTION

Matrix metalloproteinases (MMPs) comprise a family of approximately 25 zinc-dependent endopeptidases. While originally believed to primarily serve to remodel the extracellular matrix (ECM), MMPs are now understood to have a wide variety of substrates and functions, including activation of bioactive signaling molecules or other proteases and participating in transduction of cellular signaling pathways [1, 2]. MMP-14, also known as membrane type-1 MMP (MT1-MMP), is a membrane bound MMP overexpressed in most cancer cells which is correlated to poor patient prognosis [2, 3]. MMP-14 has also been demonstrated to promote cell migration and invasion [3, 4], angiogenesis [5, 6], and metastasis [7, 8]. Despite the prominent role of MMP-14 in driving cancer growth and aggression, the mechanisms by which MMP-14 are regulated remain poorly described. Transcription factors Egr-1 and Hif-2α as well as miRNA-181 have been found to be involved in regulation of MMP-14 expression [9–11]. Perhaps most notably though, regulation by transcription factor Sp1 has been identified by several groups as the primary factor regulating expression of the MMP-14 gene [12, 13]. In normal, non-cancerous adult tissue, Sp1 levels typically remain constant and do not fluctuate [14]. Despite this, MMP-14 levels are generally repressed. If Sp1 is indeed the primary transcription factor responsible for MMP-14 expression, other regulatory molecules must then have a critical role to keep MMP-14 levels in check when the body is in homeostasis.

While MMP-14 expression in human oral cancer specimens has been demonstrated by immunohistochemistry (IHC) to occur in the absence of p53 [15] and several studies have demonstrated that p53 status directly correlates with the invasiveness of tumors [16, 17], a direct or causative association between these proteins has yet to be established. While approximately 50% of all cancers have mutated p53, the gene for the so-called “guardian of the genome” remains unaffected in the other half. Despite retention of the wild type alleles, these cancers can still grow, degrade the basement membrane, and metastasize. It has recently been demonstrated that cytokine signaling via signal transduction protein gp130 in inflamed tumor microenvironments can downregulate p53 protein levels [18, 19]. Specifically, interleukin-6 (IL-6) and its closely related cousin leukemia inhibitory factor (LIF) have been implicated. Interleukin-6 is one of the most well-characterized pro-inflammatory cytokines found in tumors and has been shown to drive tumor growth and proliferation [20], stimulate angiogenesis [21], lead to chemoresistance [22], promote epithelial-to-mesenchymal transition [23], and increase the rate of metastasis [24]. It is estimated that at least 20% of cancers occur as a direct result of infection and chronic inflammation. Furthermore, the remaining 80% of these cancers often come to be highly infiltrated with inflammatory cells and exhibit high levels of cytokines within the tumor microenvironment [25].

In this study, we identified that not only can p53 repress MMP-14 expression, but that interleukin-6 signaling can decrease p53 levels and cause a concomitant increase in MMP-14 levels. We show that inflammatory cytokine signaling increases degradation of p53 to increase Sp1-mediated transcription of MMP-14. We also demonstrate that interleukin-6 promotes MMP-14-mediated invasion and metastasis using *in vitro* and *in vivo* experimental models. Our data highlight a functional role for interleukin-6 in cancer dissemination via MMP-14 and pose a new rationale for therapeutically targeting the IL-6 signaling pathway in cancer.

## RESULTS

### p53 downregulates MMP-14 expression and functions

**Figure 1:**
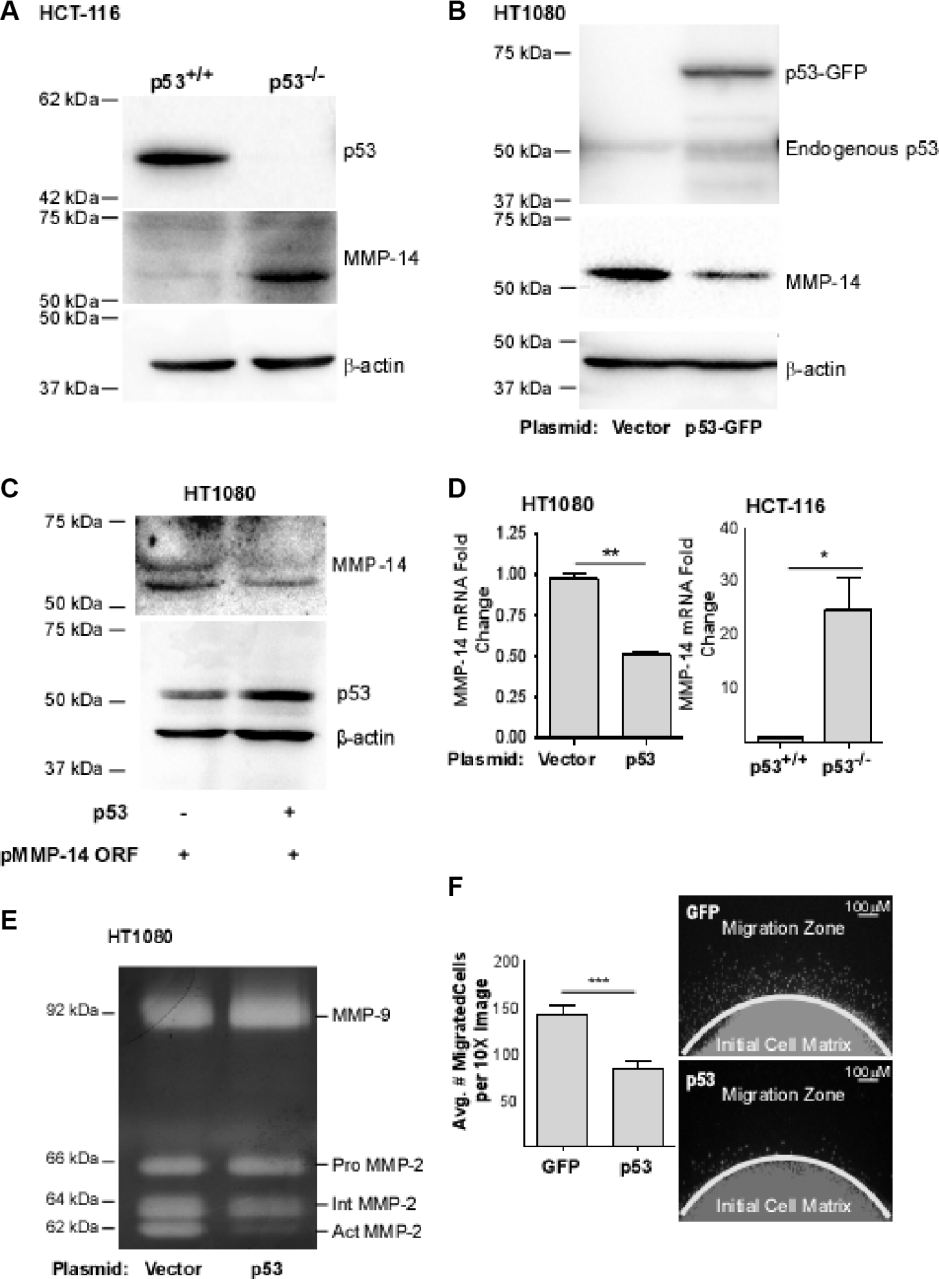
p53 expression is inversely correlated with MMP-14 levels (**A**) Western blotting was performed in p53 wild-type (p53^+/+^) and p53 null (p53^−/−^) HCT-116 cells using anti-p53 and anti-MMP-14 antibodies. β-actin was used as a loading control. MMP-14 was only detected in p53^−/−^ HCT-116 cells, but not in p53^+/+^ HCT116 cells. (**B**) Ectopic expression of p53-GFP in HT-1080 cells results in a decrease of MMP-14 levels as demonstrated by Western blotting analysis using anti-p53, anti-MMP-14 antibodies. β-actin was used as a loading control. (**C**) Co-expression of MMP-14 cDNA encoding the open reading frame of MMP-14 driven by its native promoter (pMMP-14 ORF) with p53 cDNA leads to decreased MMP-14 expression compared to vector control when examined by a Western blotting using corresponding antibodies. β-actin was used as a loading control. (**D**) Total RNA extracted from HT-1080 cells transfected with p53 or control cDNA and p53^+/+^ and p53^−/−^ HCT-116 cells was examined by real time RT-PCR using MMP-14 primers. HPRT-1 was used as a normalization control. An inverse correlation between p53 expression and MMP-14 levels was observed in gene transcriptional levels. (**E**) Gelatin zymography was employed to examine functional MMP-14 in terms of proMMP-2 activation. Ectopic expression of p53 in HT-1080 cells attenuates MMP-14-mediated proMMP-2 activation. proMMP-2: latent form of MMP-2; IntMMP-2: intermediate form of MMP-2; and act MMP-2: fully activated MMP-2. (**F**) Dot migration assay was conducted to examine the effect of p53 on cancer cell migration. HT1080 cells transfected with vector control and p53 cDNA were mixed with collagen type I and dotted onto a 96-well plate. After a 24 hours incubation, the cells were fixed and stained with DAPI for nuclear staining (right panel) followed by microscopic counting of migrated cells (left panel). *p < 0.05, **p < 0.01, ***p < 0.001.

MMP-14 is frequently overexpressed in cancer and has been shown to play a critical role in tumor growth and metastasis. While several reports have suggested a correlation between p53 status and MMP-14 expression [15–17], a direct link between the two has not been established. To determine the relationship between p53 and MMP-14 expression, we first surveyed a genetically engineered strain of the human colon cancer cell line HCT-116 in which the p53 gene was permanently knocked out (HCT-116 p53^−/−^), and compared the results with wild-type HCT-116 (HCT-116 p53^+/+^) cells. Surprisingly, an inverse correlation between p53 and MMP-14 expression was observed when examined by Western blotting analysis using corresponding antibodies (Figure 1A). This observation led us to further characterize the effect of p53 on regulation of MMP-14 expression. Human fibrosarcoma HT1080 cells, which endogenously express high levels of MMP-14, were employed to ectopically overexpress p53. When p53-GFP chimeric cDNA was transiently transfected into HT1080 cells, endogenous MMP-14 expression was reduced as compared to vector cDNA control (Figure 1B). To substantiate these observations, we cloned the human MMP-14 promoter from the genomic DNA of HT1080 cells and the promoter was placed at the 5′ end of a myc-tagged MMP-14 construct consisting of the open reading frame (named pMMP-14 ORF). When pMMP-14 ORF was co-transfected with p53 or vector control, p53 significantly reduced MMP-14 expression (Figure 1C). In addition, this demonstrates that transfection of wild-type p53 results in reduction of MMP-14 expression, ruling out the artificial effect by p53 and GFP fusion (Figure 1C). Both pro- and active forms of MMP-14 (60 and 57 kD, respectively) can be observed in our blots.

To determine if the regulation of MMP-14 by p53 occurred at the transcriptional level, real-time RT-PCR for the mRNA of MMP-14 in HCT-116 p53^+/+^ cells versus HCT-116 p53^−/−^ cells and HT-1080 cells transiently overexpressing p53 or vector control was performed. Our real-time RT-PCR data substantiates the Western blot data and demonstrates that p53 expression is associated with significantly decreased MMP-14 mRNA (Figure 1D).

To further determine whether reduced MMP-14 expression by p53 decreases functional MMP-14, we employed a MMP-14 functional assay by monitoring latent MMP-2 (proMMP-2) activation. proMMP-2 is a secretory MMP in which the prodomain is partially cleaved by functional MMP-14 to produce an intermediate form (IntMMP-2) which then becomes fully activated MMP-2 (ActMMP-2) [26]. The conditioned media from HT-1080 cells overexpressing p53 or vector control were examined by gelatin zymography. Consistent with the protein expression level of MMP-14, ectopic expression of p53 resulted in decreased proMMP-2 activation as compared to vector control, whereas proMMP-9 is unaffected (Figure 1E).

Because MMP-14 has been shown to increase cancer cell migration independently of its catalytic function [27], a two-dimensional cell migration assay was therefore used to determine if the p53-mediated downregulation of MMP-14 results in decreased migration of HT-1080 cells. As expected, cells overexpressing p53 migrated significantly less than the control cells (Figure 1F). The phenotypes observed are not due to differences in cell viability caused by differences in p53 levels. In HT-1080 cells transient transfection of p53 did not induce apoptosis within the time course of the experiments as evidenced by apoptosis assay using Annexin V as a marker followed by flow cytometry analysis (Supplementary Figure S1A). Similarly, loss of the p53 gene did not significantly affect cell viability in HCT-116 cells as measured using the MTT viability assay (Supplementary Figure S1B). Our data suggest that wild-type p53 affects MMP-14 gene expression leading to reduced function of MMP-14.

### p53 represses MMP-14 promoter activity

**Figure 2:**
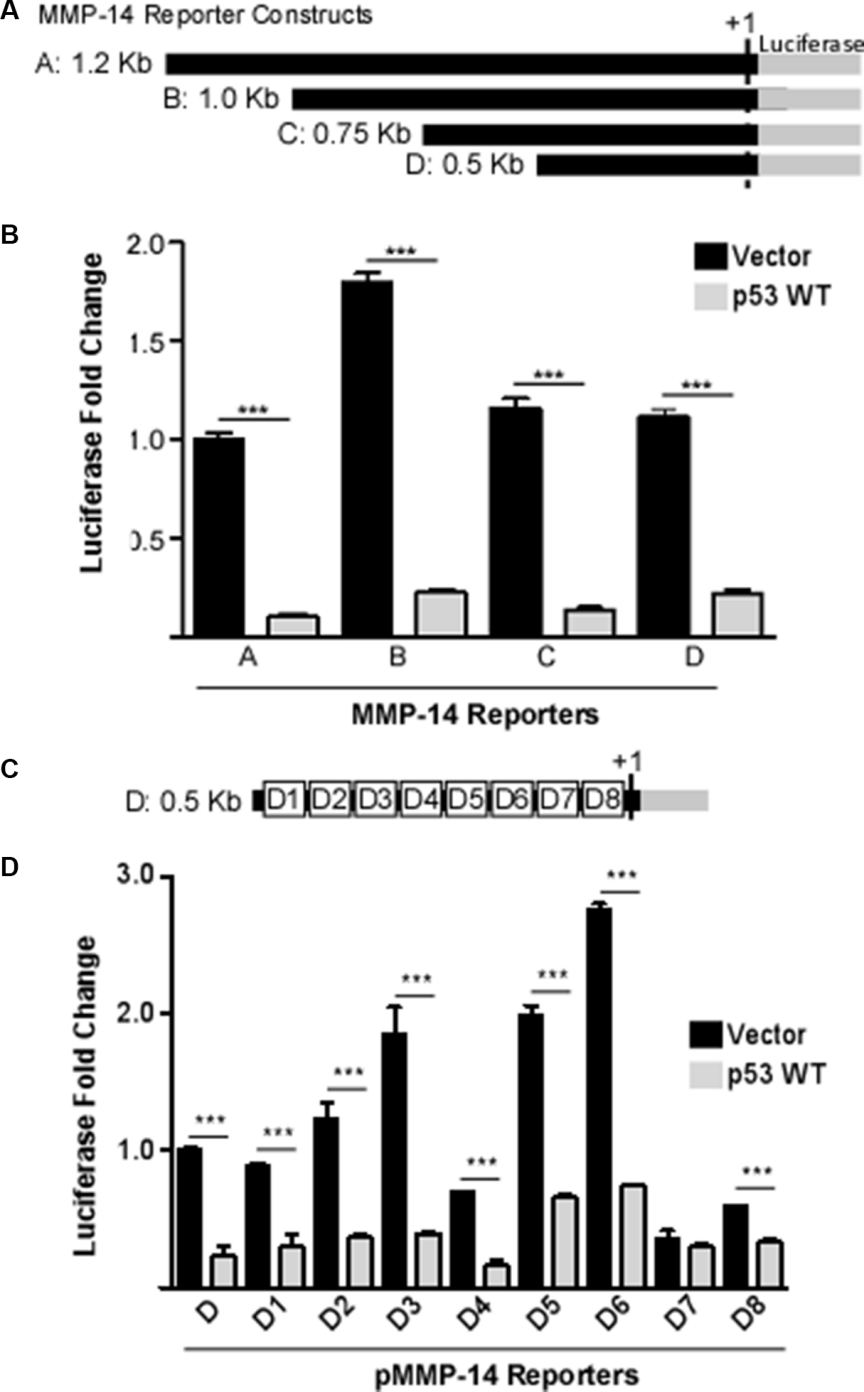
Identification of p53 response element with the MMP-14 promoter (**A**) A schematic diagram of the full length and truncated MMP-14 promoters termed truncation A through D. (**B**) Co-transfection of p53 cDNA with pMMP-14-Luc demonstrates by luciferase assay that p53 decreases MMP-14 promoter induction. As the shortest truncation, truncation D, also has decreased promoter induction by p53 compared to vector control, region D is determined to contain the p53 regulatory response element. *Renilla* luciferase was used as a normalization control. (**C**) A schematic diagram of deletion mutants of approximately 50 bp within the region D. (**D**) Co-expression of p53 cDNA with pMMP-14-Luc at deletion 7 no longer attenuates promoter induction compared to vector control, indicating D7 contains the p53 regulatory response element. *Renilla* luciferase was used as a normalization control. ***p < 0.001.

To dissect the molecular mechanism underlying p53-regulated MMP-14 expression, we characterized the effect of p53 on MMP-14 promoter activity. A bioinformatics approach employing two different promoter-prediction programs (Genomatix Model Inspector and Promoter- Prediction Server at Duke University) was conducted to identify the promoter region of MMP-14. We subsequently utilized PCR amplification of the promoter using genomic DNA derived from HT1080 cells as a template. A 1.2 kb fragment of the deduced promoter including the first exon was amplified and cloned into a pGL3-basic vector lacking a promoter and including the firefly luciferase reporter gene. Since p53-mediated reduction of MMP-14 promoter activity may be through a direct or an indirect effect on the MMP-14 promoter, 5′ deletion analysis was conducted. Parts of the MMP-14 promoter were sequentially deleted starting from the 5′-end to generate 3 different deletion mutants of different lengths (Figure 2A). The deletion constructs were co-transfected with p53 or vector control into COS-1 cells followed by the Dual Luciferase assay. p53 represses all 3 deletion mutants similar to full-length MMP-14 promoter as compared to vector control (Figure 2B), suggesting that the response element of p53 within the MMP-14 promoter may lie within the 500 bp region of the promoter immediately upstream of the transcription start site (+1). To further narrow down the region containing the p53 response element, sequential deletions of approximately fifty base pairs starting at the 5′-end of the 0.5 kb MMP-14 promoter were generated (Figure 2C). As evidenced by the results of the luciferase assay, only deletion of region seven (D7, nt ^−^94 to nt ^−^44) abolished the ability of p53 to negatively affect the promoter activity compared to the control (Figure 2D).

### p53 regulates MMP-14 promoter activity by competitively interacting with the transcription factor Sp1 at the MMP-14 promoter

**Figure 3:**
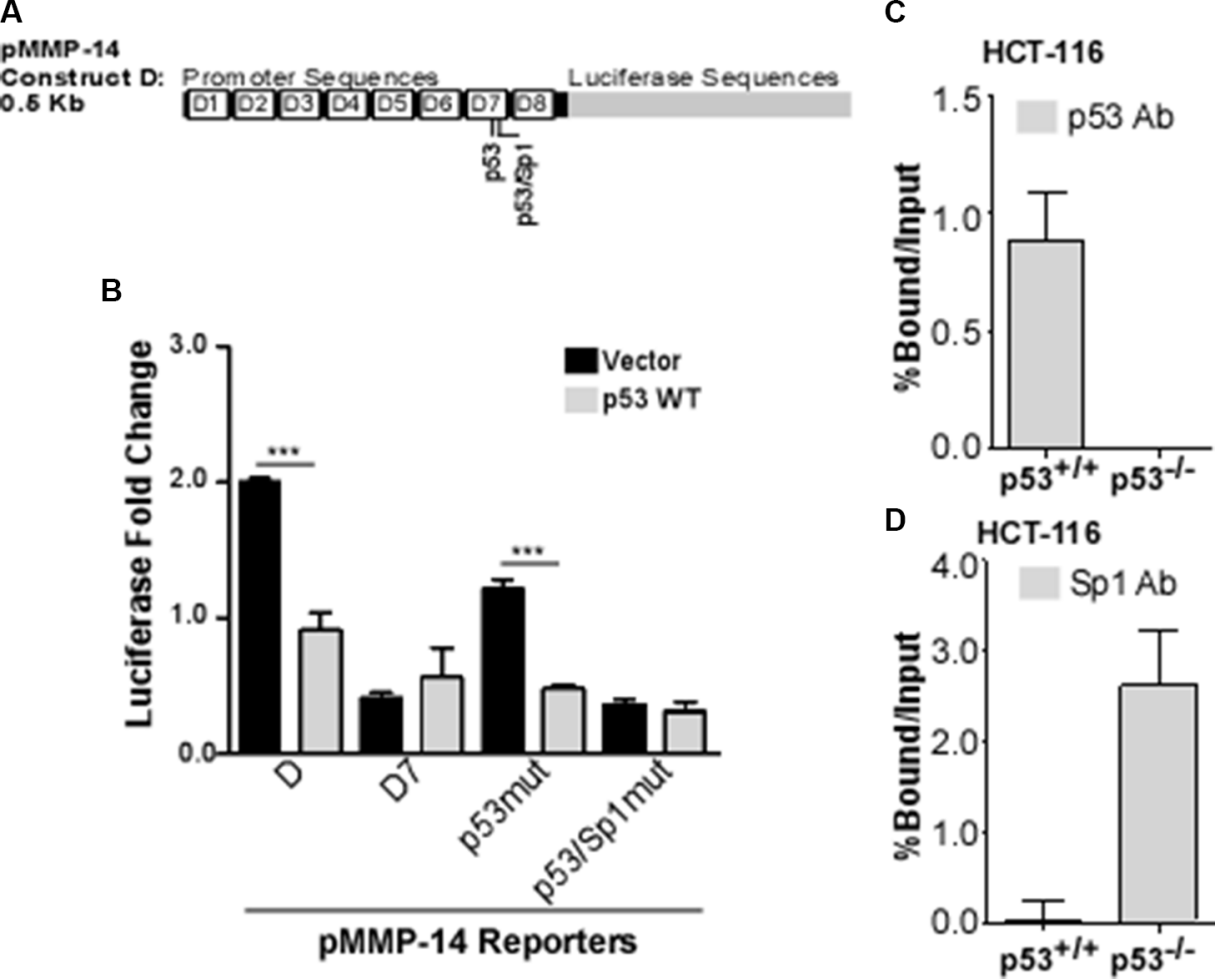
p53 and Sp1 bind competitively at the MMP-14 promoter (**A**) Bioinformatic analysis identifies two potential p53 binding sites within deletion 7, the second of which is also a predicted Sp1 binding site. (**B**) Reporter gene assay was performed in COS-1 cells co-transfected with MMP-14 reporters for truncation D, D7, and mutated p53 or p53/Sp1 response elements (termed p53mut or p53/Sp1mut, respectively) along with either vector control or wild type p53 cDNAs followed by a luciferase assay. *Renilla* luciferase was used as a normalization control. Mutation at the second p53 binding element no longer responds to p53 expression. (**C**) ChIP-qPCR assay was performed using a p53 antibody to precipitate DNA from p53 wild-type (p53^+/+^) and p53 null (p53^−/−^) HCT-116 cells. Real time RT-PCR was then conducted using primers specific for MMP-14 promoter. MMP-14 promoter is precipitated by p53 antibody in HCT-116 (p53^+/+^) cells, whereas none is precipitated in p53^−/−^ cells. (**D**) ChIP-qPCR was performed in HCT-116 cells with wild type or null p53 expression using anti-Sp1 antibody followed by a real time RT-PCR for MMP-14 promoter region. IgG and input samples were used as normalization controls. Results are expressed as percent of input. ***p < 0.001.

Using the bioinformatics analysis tool PROMO as previously described [28, 29], we identified 3 potential p53 response elements within the MMP-14 promoter: -nt1137 to -nt1130, -nt74 to -nt68, and -nt66 to -nt59. The first p53 response element was in the antisense orientation; however, this region was already ruled out as required for p53-mediated regulation of MMP-14 (refer to Figure 2A). When the second and third deduced p53 response elements were individually mutated, only the third predicted response element, -nt66 to -nt59 failed to respond to p53 regulation (Figure 3A and 3B). Interestingly, this p53 response element is partially overlapped with a predicted Sp1 binding site, suggesting p53 and Sp1 could competitively bind to the MMP-14 promoter (Figure 3A).

Sp1 has been previously reported to positively regulate MMP-14 expression [13, 30, 31]. Separately, studies have demonstrated that p53 can bind to promoters to repress Sp1-mediated transcription by blocking access of this transcription factor to the promoter [32–34]. To examine if p53 and Sp1 competitively bind to the MMP-14 promoter at -nt66 to -nt59, we performed chromatin immunoprecipitation (ChIP) coupled with quantitative real-time PCR to study p53 and Sp1 binding to the MMP-14 promoter. Notably, a significant amount of DNA is precipitated by the p53 antibody at the p21 promoter in p53 wild type cells only, while minimal or no DNA is precipitated from the p53 null cells or by the Sp1 antibody, validating our ChIP system (Supplementary Figure S2A). Additional validation demonstrated that minimal DNA is precipitated by either p53 or Sp1 antibodies from the MMP-14 promoter in a region outside the predicted binding sites, as expected (Supplementary Figure S2B). In the HCT-116 cells that express wild type p53, DNA at the MMP-14 promoter was precipitated with the p53 antibody but not in the p53 null HCT-116 cells (Figure 3C). However, in the p53 null cells, the amount of DNA at the MMP-14 promoter precipitated by the Sp1 antibody displayed 3 times more compared to that precipitated from the p53 wild type cells (Figure 3D). Importantly, no significant differences are observed in Sp1 levels after over-expression of wild type p53 in HT-1080 cells or loss of the p53 gene in HCT-116 cells (Supplementary Figure S3).

### Interleukin-6 decreases p53 and causes a concomitant increase in MMP-14 expression to increase metastases and metastatic growth *in vivo*

**Figure 4:**
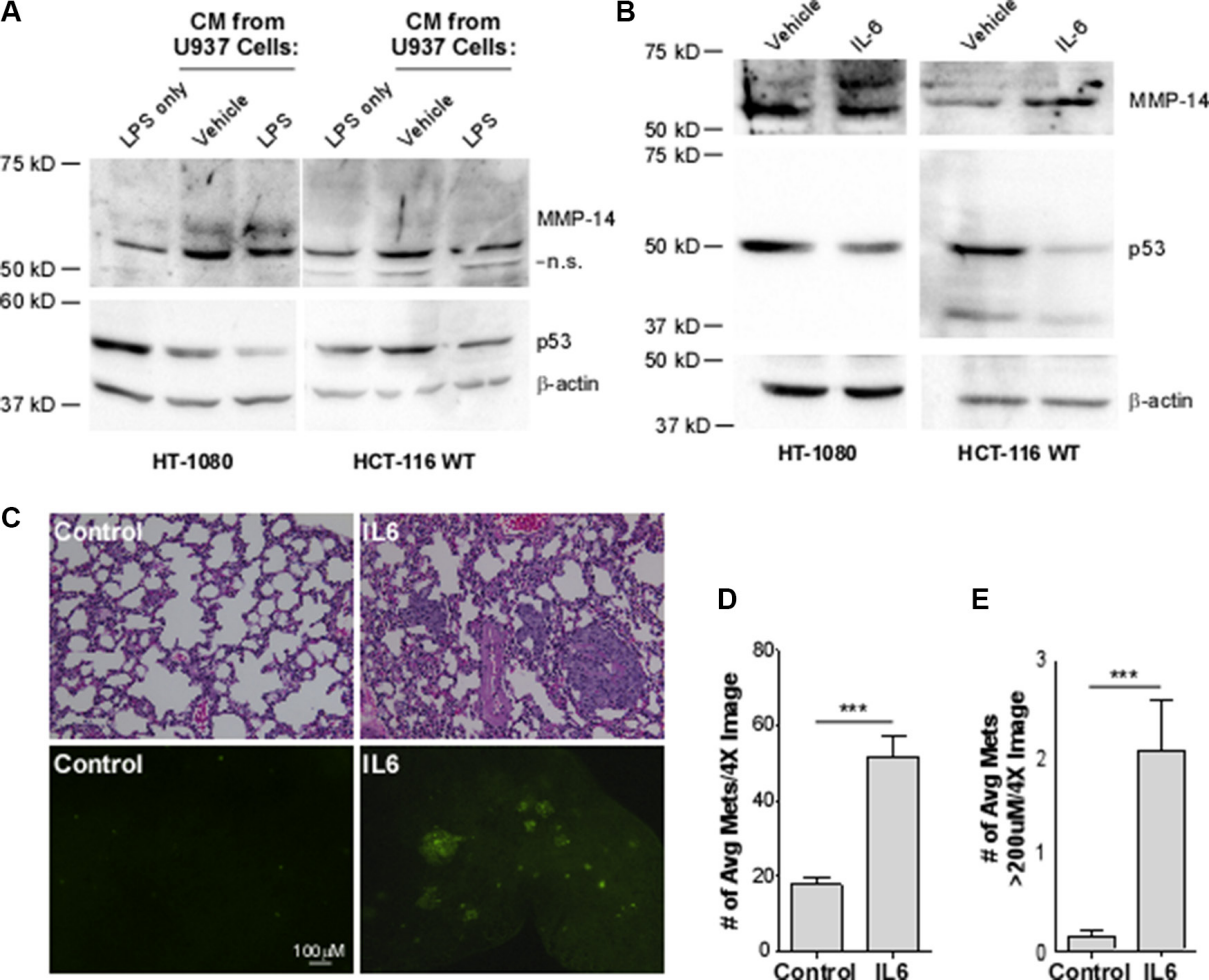
Interleukin-6 decreases p53 levels, leading to a concomitant increase in MMP-14 expression in HT-1080 and HCT-116 WT cells and drives metastasis and growth of HT-1080 cancer cells *in vivo* (**A**) Western blotting analysis was performed on HT-1080 and HCT-116 p53 wild type (WT) cells in the presence of LPS or with conditioned media (CM) from U937 macrophage cells that were activated with LPS to induce cytokine secretion or with water as vehicle control for 24 hours. While CM from U937 cells increase MMP-14 levels, only after macrophage activation was a decrease in p53 observed using anti-p53 and anti-MMP-14 antibodies. β-actin was used as a loading control. (**B**) Western blotting analysis was performed on HT-1080 and HCT-116 cells treated with 50 ng/mL recombinant IL-6 or 1% BSA vehicle control for 24 hours followed by Western blotting. A decrease in p53 was observed along with a concomitant increase in endogenous MMP-14 using the corresponding antibodies. β-actin was used as a loading control. (**C**) H&E staining of paraffin-embedded sectioned lung tissue sections (top panel) and the presence of GFP lesions on the gross lung tissue (bottom panel) indicates increased formation of metastases. The number (**D**) and size (**E**) of GFP-positive lesions per image are significantly increased in mice which received IL-6-treated cells (*n* = 9) compared to control (*n* = 10). Lesion size was determined using Nikon Imaging Software. ***p < 0.001.

Inflammation is an important hallmark of cancer, known to drive cancer progression and aggression. It has recently been demonstrated that interleukin-6 (IL-6) can downregulate p53 protein levels [18]. To explore a potential cascade of IL-6 and p53 in driving MMP-14 expression, we initially obtained cytokines, which include IL-6, released from the human monocytic precursor cell line U937. Using a two-step induction process of U937 cells sequentially treated with PMA and LPS, IL-6 is released from differentiated monocytes or mature macrophages by LPS [35, 36]. The conditioned medium containing IL-6 was then incubated with both HCT-116 and HT-1080 cells followed by Western blotting analysis. The conditioned media from U937 macrophages doubled the levels of MMP-14 in both cells lines. Stimulation of the U937 cells, however, with LPS led to a 50% decrease in p53 levels (Figure 4A, Supplementary Figure S4).

Since the conditioned medium collected from the mature macrophages contains cytokines in addition to IL-6, we next treated the cells using pure recombinant IL-6 protein in order to determine the role of IL-6 in regulation of p53 and MMP-14. HT1080 cells were treated with recombinant IL-6 followed by Western blotting analyses for p53 and MMP-14 using corresponding antibodies. Treatment of HT1080 cells with IL-6 decreased p53 protein levels leading to increased MMP-14 expression (Figure 4B, left panel). In agreement with the observation from HT1080 cells, treatment of wild-type HCT116 with recombinant IL-6 also resulted in decreased p53 and increased MMP-14 (Figure 4B, right panel). These results indicate that MMP-14 is regulated through IL-6-p53 cascade.

We next examined if upregulated MMP-14 by IL-6 contributes to promoting cancer dissemination. Given the limitation of the *in vivo* model system for constant presence of localized IL-6, an experimental metastasis model was chosen in which HT1080 cells were treated with IL-6 or vehicle control and then injected via tail vein. We first generated stable HT1080 cells expressing GFP to facilitate visualization of lung metastatic tumor colonization. After 10 days post-injection of the cells pre-treated with IL-6 or vehicle control for 24 hours, animals were sacrificed and the lungs were microscopically examined for tumor nodules based on GFP marker and hematoxylin and eosin (H&E) staining. The lungs of animals injected with the IL-6-treated cells were better colonized by tumor cells and had more fibrotic lesions compared to the lungs of animals receiving vehicle-treated cells (Figure 4C). Analysis of the gross tissue shows increased GFP-positive colonies, representing metastases to the lungs. Furthermore, IL-6 treatment significantly increased both the number and size of the metastases present on the lungs compared to vehicle control (Figure 4D and 4E). Our study collectively suggests that MMP-14 is negatively regulated by wild-type p53, and MMP-14 is upregulated in the tumor microenvironment as a result of IL-6-mediated suppression of p53 levels.

### Interleukin-6 decreases p53 expression by increasing its degradation

**Figure 5:**
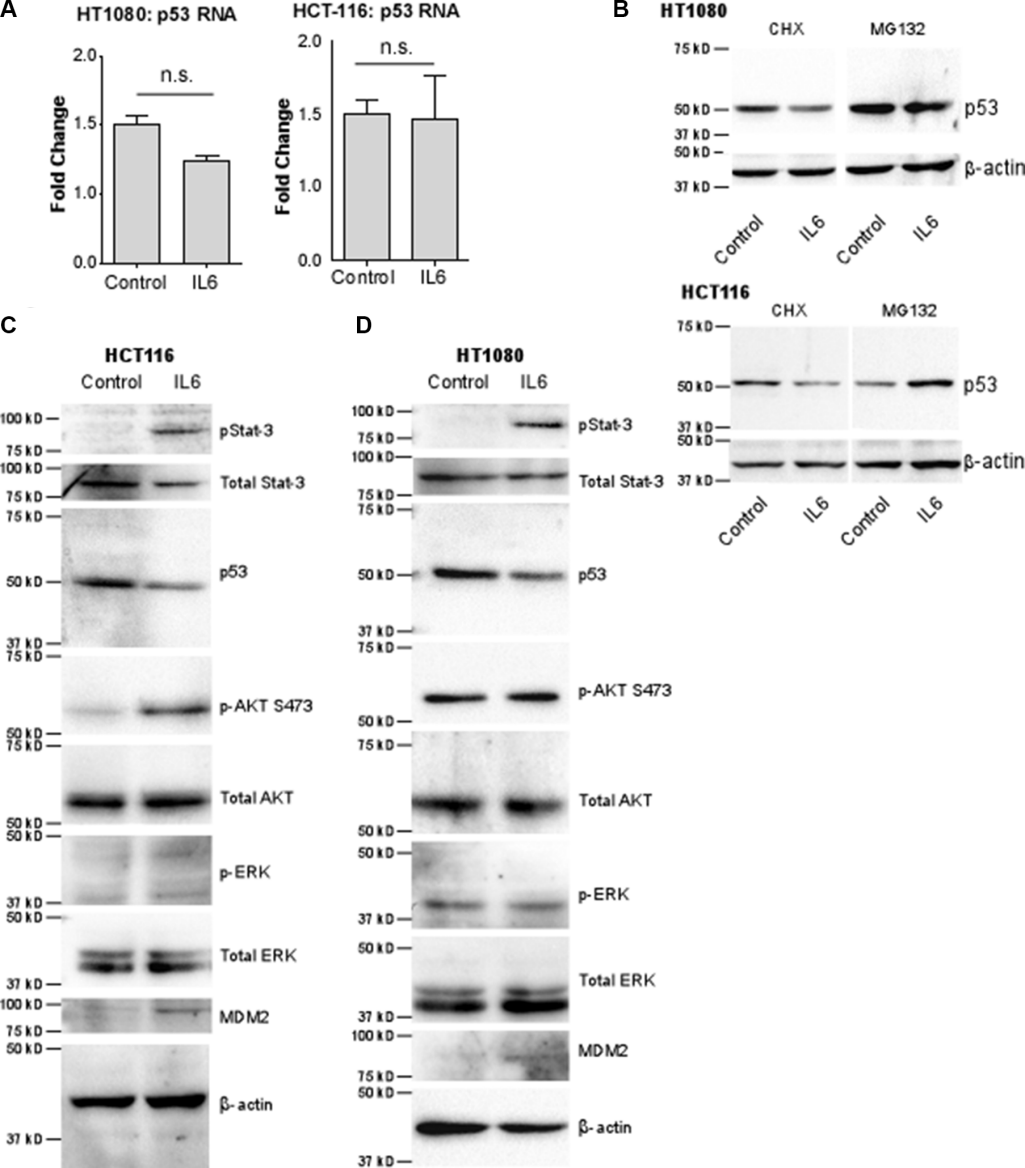
The decrease of p53 by IL-6 is due to increased rate of degradation by E3 ubiquitin ligase MDM2 via cell-specific signaling pathways (**A**) p53 mRNA levels normalized to HPRT-1 were analyzed by real time RT-PCR. p53 mRNA levels do not significantly change in either HT-1080 or HCT-116 cells after stimulation with IL-6. (**B**) Western blotting of HT-1080 and HCT-116 cells using an anti-p53 antibody demonstrates that inhibition of protein synthesis by 20 ug/mL cycloheximide (CHX) in both cells lines has no effect on the decrease in p53 protein stimulated by IL-6. However inhibition of proteasomal degradation by 10 uM MG-132 completely blocks downregulation of p53 and stabilizes p53 protein levels, indicating IL-6 regulates p53 levels by increasing its degradation. β-actin was used as a loading control.(**C**) In HCT-116 cells, IL-6 stimulation activates p-Stat3 and p-AKT pathways compared to vehicle control. Furthermore, MDM2 levels are increased whereas p53 levels are decreased as evidenced by western blotting using the corresponding antibodies. The MAP-K pathway is not activated by IL-6, as evidenced by the lack of ERK 1/2 phosphorylation in response to IL-6. (**D**) In HT-1080 cells were treated as in C and analyzed by Western blotting. In this cell line, the p-Stat3 pathway only is activated, leading to increased MDM2 levels and decreased p53 levels.

IL-6 has been reported to regulate p53 protein levels [22]. However, the regulatory mechanism remains elusive. We next sought to determine the mechanism by which IL-6 downregulates p53. It is known that p53 is mainly regulated post-translationally [37]. As anticipated, treatment of cells with IL-6 did not significantly decrease p53 mRNA levels in both HT1080 and HCT116 p53^+/+^ cells, suggesting IL-6 regulates p53 post-translationally (Figure 5A). To test this possibility, HT-1080 cells and HCT-116 p53^+/+^ cells were treated with a protein synthesis inhibitor, cycloheximide, or a proteasome inhibitor, MG-132, before IL-6 or vehicle control treatment. While treatment of both cell lines with cycloheximide had no effect on the decrease of p53 caused by IL-6, MG-132 abolished the effect of IL-6 on downregulation of p53 protein level in both cell lines (Figure 5B). Together, this suggests that IL-6 exerts its effect on p53 expression by increasing proteasomal degradation of p53.

Because MDM2 is the known E3 ubiquitin ligase for p53, we next examined MDM2 levels after treatment with IL-6. Western blotting analyses demonstrate that IL-6 treatment increases MDM2 expression in both HT-1080 and HCT-116 cells (Figure 5C and 5D), supporting our hypothesis that IL-6 leads to p53 degradation via MDM2.

**Figure 6:**
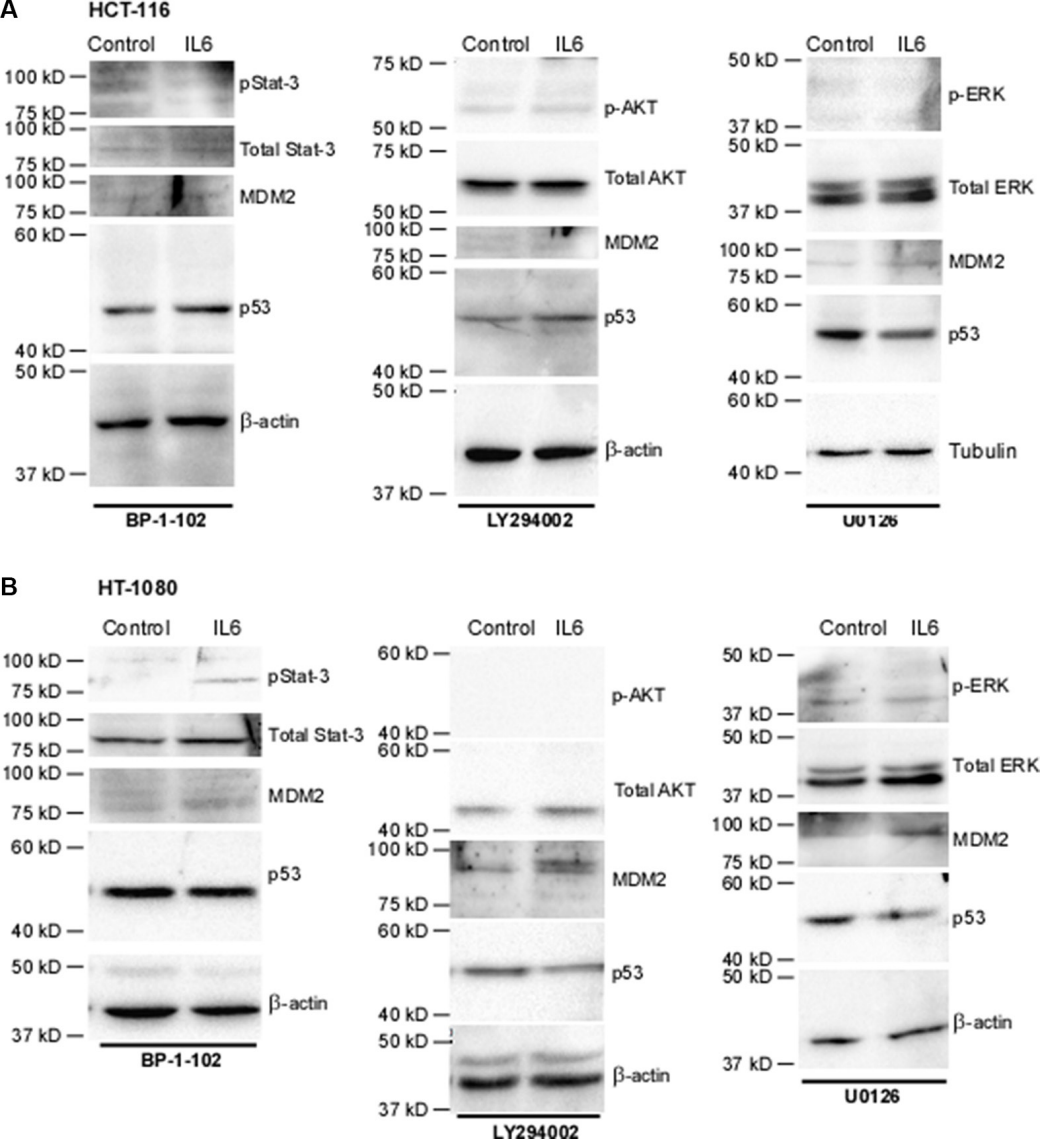
Inhibition of signaling pathways identifies key mediators of the IL-6-driven decrease in p53 in a cell-specific manner (**A**) In HCT-116 cells, treatment with p-Stat3 inhibitor BP-1-102 at 5 ug/mL abrogates the down-regulation of p53 by IL-6 as well as the increase in MDM2. Successful inhibition of PI3K, as observed via lack of phosphorylation of effector AKT, using 20 uM LY294002 abrogates IL-6-downregulation of p53 as well as the increase in MDM2. Inhibition of MEK, as observed via lack of phosphorylation of effector molecules ERK 1/2, with 20 uM U0126 has no effect on IL-6-driven regulation of p53 or MDM2, as expected. β-actin was used as a loading control. (**B**) Treatment of HT-1080 with BP-1-102 abrogates the down-regulation of p53 by IL-6 as well as the increase in MDM2. Neither treatment with LY294002 or U0126 in these cell lines upsets the effects of IL-6 on MDM2 levels, as expected. U0126 does not affect IL-6 downregulation of p53. However, due to the effects of LY294002 on blocking nuclear transport of MDM2 the p53 levels do not decrease in response to IL-6 in this condition. β-actin was used as a loading control.

We then sought to decipher the signaling pathway by which IL-6 leads to increased MDM2 and thus decreased p53 levels. The classical signaling pathway through which IL-6 functions is via binding to the gp130 Janus associated kinase (gp130-JAK) receptor and stimulating phosphorylation of Stat3. p-Stat3 then translocates to the nucleus where it acts as a transcription factor [38]. Treatment of both HCT-116 cells (Figure 6A) and HT-1080 cells (Figure 6B) with the phospho-Stat3 inhibitor BP-1-102 prior to IL-6 treatment attenuated the IL-6-mediated increase in MDM2 levels and thus abrogated the ability of IL-6 to decrease p53 levels (refer to Figure 5C and 5D).

IL-6 is also known to be capable of activating the MAP-kinase pathway and the PI3k/AKT pathway. HCT-116 and HT-1080 cells were serum-starved and then pre-treated with either LY294002, a PI3K inhibitor, or U0126, a MEK inhibitor, followed by treatment with IL-6. In HCT-116 cells (Figure 5C), treatment of IL-6 increased phosphorylation of AKT, an effector molecule of the PI3K pathway. Inhibition of AKT activity abrogated the downregulation of p53, an expected result given that phosphorylation of MDM2 by AKT is required in order for MDM2 to translocate to the nucleus where it degrades p53 (Figure 6A). Surprisingly, inhibition of PI3K phosphorylation also abrogated the effect of IL-6 on total MDM2 levels. This suggests a previously unknown mechanism of regulation of MDM2 expression in HCT-116 cells. Interestingly, we also found that inhibition of phospho-Stat3 blocks phosphorylation of AKT (Supplementary Figure S5), suggesting that the activation of phospho-AKT is downstream of phospho-Stat3 and not necessarily independent of it. In HT-1080 cells (Figure 5D), phosphorylation of AKT was not increased by IL-6, although similar to HCT-116 cells inhibiting p-AKT did abrogate the IL-6-mediated downregulation of p53 as expected without affecting IL-6 upregulation of total MDM2 levels (Figure 6B). Neither cell line demonstrated any activation of p-ERK nor was IL-6-mediated downregulation of p53 or upregulation of MDM2 affected by inhibition of this pathway (Figure 5C and Figure 5D). We therefore conclude that the MAP-kinase pathway does not contribute to IL-6 regulation of p53 in these cell lines.

## DISCUSSION

**Figure 7:**
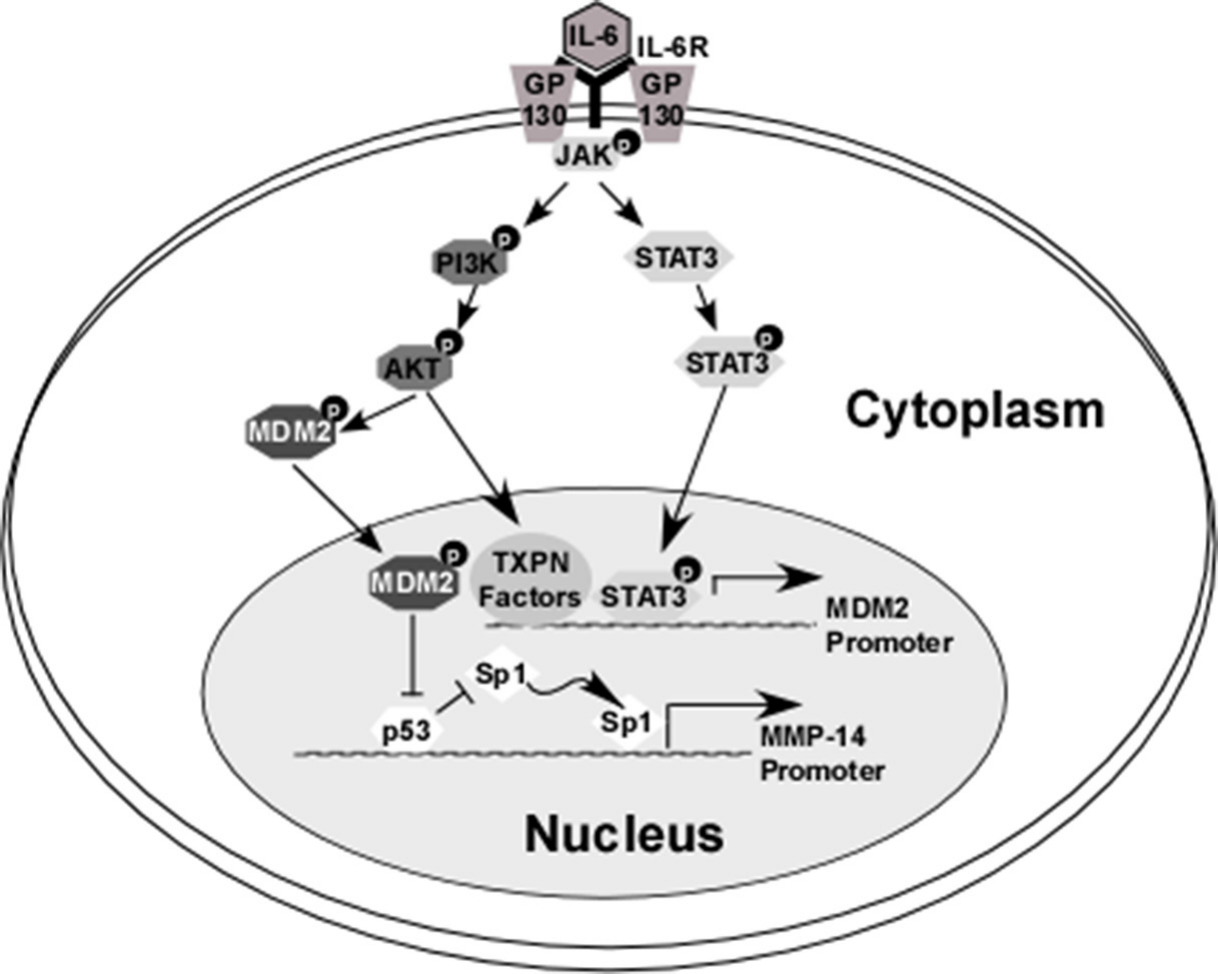
Overview of the mechanism by which IL-6 downregulates p53, leading to increased MMP-14-expression IL-6 binds to its receptor which then forms a complex with GP130. This leads to activation of JAK kinase, which in turn activates STAT3 and AKT (via the PI3K pathway). Phosphorylation of STAT3 leads to translocation of this molecule to the nucleus where it serves as a transcription factor and induces expression of E3 ubiquitin ligase MDM2. Similarly, IL-6-induced phosphorylation of AKT in HCT-116 (but not HT-1080) cells leads to increased expression of MDM2 levels. Furthermore, p-AKT induces the translocation of cytosolic MDM2 molecules to the nucleus. A primary target of MDM2 is p53; ubiquitination and subsequent degradation of p53 at the MMP-14 promoter allows for transcription factor Sp1 to bind to the MMP-14 gene and increase its expression.

In this study, we demonstrate for the first time a role for the tumor suppressor p53 in down-regulating MMP-14 expression. We then demonstrate that p53 regulates MMP-14 expression at the transcriptional level by binding to the promoter region and competitively blocking binding of the known MMP-14 transcription factor Sp1. We also identify the mechanisms by which IL-6 can increase the rate of proteasomal degradation of p53, allowing for the Sp1 binding site to become exposed and thus MMP-14 levels to increase (Figure 7). Importantly, our work shows that IL-6 confers increased invasive and metastatic potential even to cancer cells which are p53 wild type.

p53, although largely known for its transcriptional capabilities, performs a multitude and diverse spectrum of activities within cells. Not only can p53 bind to DNA in a sequence specific manner to increase gene expression, but it can also function to repress gene expression [39]. Three mechanisms of p53-mediated repression have been proposed: (1) interference of the transcriptional complex assembly on the promoter, (2) interference of assembly of the basal transcriptional machinery itself which may be DNA-independent, and (3) recruitment of chromatin-modifying factors to reduce promoter accessibility [39]. Our research identifies repression of MMP-14 expression by p53 as belonging to the first category. While several groups have demonstrated previously that the transcription factor Sp1 regulates MMP-14 expression [9, 12], a relationship of p53 to MMP-14 expression has never before been clearly described. Analysis at the promoter indicates that p53 competes for binding with Sp1, and once bound sterically blocks Sp1 binding. It is therefore of little surprise that nearly all cancer cell lines expressing high levels of MMP-14 are either p53 low, p53 null, or harbor a mutation in p53.

While IL-6 has previously been linked to decreased p53 levels, the mechanism of how this regulation occurs has not been thoroughly investigated. Herein, we demonstrate that IL-6 activates via phosphorylation transcription factor Stat3 which increases MDM2 levels. Of note, our data also implicates a role for AKT in this pathway but via a novel activation mechanism. Previous work by others has concluded that IL-6 signaling can lead to phosphorylation and activation of AKT via the PI3k pathway [40]; however, our data demonstrates that activation of AKT in HCT-116 cells can actually occur as a result of Stat3 activation (Supplementary Figure S4). Due to the fact that IL-6 does not activate AKT in HT-1080 cells, it is likely that different cell lines or different cancers may activate different pathways in response to IL-6. This supposition is supported by the work of two other groups who have investigated a mechanism by which inflammation leads to downregulation of p53. Yu et al. found that LIF, a member of the IL-6 family, also leads to p53 downregulation via activation of Stat3 with a subsequent increase in MDM2 levels [19], although they did not investigate AKT in their study. Brighenti et al. on the other hand convincingly describe a different mechanism by which IL-6 signaling indirectly leads to decreased p53 levels. In their model, IL-6 stimulates ribosomal biogenesis, a Stat3-driven process which results in increased amounts of MDM2 available for binding p53 without increasing total MDM2 levels [41]. Furthermore, this work goes on to describe how IL-6-driven downregulation of p53 induces phenotypic changes in cells indicative of an epithelial-to-mesenchymal transition. From this work done by us and others we can extrapolate that inflammation can drive cancer progression but the mechanism by which this occurs can vary.

The data presented herein not only provide a plausible and convincing explanation for how IL-6 increases MMP-14 in cancer to drive metastasis, but implications of this research can be extended into multiple other fields of study. Indeed, pathological states in which inflammation is correlated with increased MMP-14 expression include irritable bowel disease which causes increased MMP-14 levels in the colonic mucosa [42], inflammation resulting from cardiac myopathies causing increased MMP-14 in myocardium [43], and atheroma-associated inflammation leading to increased levels of MMP-14 in vascular epithelial cells [44]. In each of these cases, the increase in MMP-14 correlates with remodeling of the tissue and/or vasculature that can lead to increased morbidity and rates of mortality. While MMP-14 is an attractive therapeutic target, attempts at inhibiting MMPs directly have failed spectacularly during clinical trials [45]. Establishing a causative link between inflammation and MMP-14 expression thus opens the door for development of therapeutics which may disrupt this signaling pathway. Several monoclonal antibodies and small molecule inhibitors of the IL-6 pathway have already been evaluated clinically for a variety of inflammatory disorders, with IL-6 receptor antagonist tocilizumab (Actemra, Genentech), a recombinant humanized monoclonal antibody, clinically approved for treatment of rheumatoid arthritis. The data presented in this paper strongly support the idea that IL-6 inhibitors may be of great benefit to cancer patients. It is also worth noting that IL-6 signaling in the tumor microenvironment is actually increased after chemotherapy and directly contributes to chemoresistance. IL-6 can turn on expression of multidrug resistance protein 1 (mdr1) [46], allow cells to evade apoptosis [22], and attenuate the antigen-presenting capabilities of dendritic cells in the tumor microenvironment to repress an immunogenic response [47]. As such, co-therapy of an IL-6 pathway inhibitor with standard chemotherapy should theoretically produce a synergistic response and vastly improve patient outcomes.

## MATERIALS AND METHODS

### Materials

Anti-p53, anti-phospho-Stat-3 and total Stat-3 antibodies were acquired from Santa Cruz Biotechnology; anti-MMP-14 antibodies were acquired from Millipore; anti-Sp1 and anti-MDM2 were acquired from Abcam; anti-myc tag was acquired from Roche; anti-phospho-ERK 1/2 and anti-total ERK antibodies were acquired from Sigma-Aldrich; anti-phospho-AKT and anti-total AKT antibodies were obtained from Cell Signaling. Anti-actin antibodies were attained from Cell Signaling Technologies and anti-IgG was obtained from Millipore for probing loading or experimental setup controls, respectively. Horseradish-peroxidase (HRP) conjugated anti-mouse and anti-rabbit secondary antibodies were acquired from Rockland Immunochemicals. Hoechst nuclear stain was acquired from Invitrogen. Proteasome inhibitor MG-132 and protein synthesis inhibitor cycloheximide were acquired from Sigma-Aldrich. Recombinant IL-6 was purchased from either Sigma-Aldrich or Gold Biotechnology. STAT3 Inhibitor XVIII, BP-1-102 and PI3K inhibitor LY294002 were acquired from Calbiochem; MEK1/2 inhibitor U0126 was acquired from Cell Signaling Technologies.

### Cell culture and transfection

All cell lines used in this study, except HCT-116 p53 −/−, were purchased from the American Type Culture Collection (ATCC, Manassas, VA) and cultured as recommended. The HCT-116 p53 −/− cell line was kindly provided to us by Dr. Ute Moll (Stony Brook University, NY, USA). HT-1080, HCT-116, and HCT-116 p53 −/− cells were cultured in DMEM media containing 10% FBS and in 5% CO_2_. U937 cells were cultured in RPMI1640 media containing 10% FBS and in 5% CO_2_. To achieve transient transfection of cells, polyethyleneimine (MW: 250 K, Polysciences) was incubated with plasmid DNA for 30 minutes at room temperature prior to addition to cells. Medium was replaced after 18 hours and assays were performed after the indicated recovery period.

### Quantitative real-time PCR

RNA from cultured cells was isolated by Trizol and reverse transcriptase (BioRad iScript cDNA Synthesis Kit) was used to generate cDNA. Quantitative real-time PCR was performed using BioRad iQ SYBR-Green Super Mix on a BioRad iQ5 Real Time PCR machine. Relative expression was calculated using the ΔΔCt method. HPRT-1 was used as an internal control. Primers used for detection can be found in Supplementary Table S1.

### Dot-based cell migration assay

HT-1080 cells were transiently transfected for 18 hours, embedded into a collagen matrix, and dotted in a 96-well plate. Solidified cell-matrix dots were overlaid with complete media. Cells were allowed to migrate for up to 8 hours, fixed, and then stained in Hoechst/PBS (1:2000). Images were captured using the previously described microscope and camera system and migration was quantified by counting nuclei using the Nikon Elements Basic Research Software analysis tools [48].

### DNA constructs and dual-luciferase assays

Construction of the 1.2 kb MMP-14 promoter luciferase reporter was performed using a PCR approach as we have previously described [49, 50]. The PCR reaction was carried out in the presence of 0.5 M GC-rich resolution buffer (Roche) and the PCR products were then cloned into pGL3 basic vector which contains the firefly luciferase reporter gene. Truncations, deletions, and scramble mutations to this construct were generated using the Q5 Site-Directed Mutagenesis Kit (New England Biolabs) as previously described [51]. Primers used can be found in Supplementary Table S1. All constructs were confirmed by DNA sequencing. To examine promoter activity, Cos-1 cells were transiently transfected with the luciferase promoter constructs, p53 or vector control, and Renilla luciferase reporter gene (1:1:0.5) using polyethylenimine for 18 hours. 8 hours post-transfection, firefly and Renilla luciferase activities were measured using the Dual-Glo Luciferase Assay system (Promega) as we have previously described [52].

### Chromatin immunoprecipitation (ChIP)

The ChIP assay was performed based on the Zymo-Spin ChIP Kit (Zymo Research) using the anti-p53 antibody or the anti-Sp1 antibody. Briefly, cellular proteins were cross-linked with chromosomal DNA by 1% formaldehyde followed by sonication. Lysates were immunoprecipitated with the indicated antibody or rabbit IgG as a control (Millipore) at 4°C overnight in the presence of protein-A agarose beads. Immunoprecipitated DNA was amplified by real-time PCR using either a pair of primers spanning the p53/Sp1 binding site within the MMP-14 promoter or primers spanning various regions indicated in Supplementary Table S2. Immunoprecipitated DNA was calculated according to the bound (immunoprecipitated chromatin)/input ratio. Primers used can be found in Supplementary Table S2.

### Mouse metastasis model

Human HT-1080 cancer cells stably expressing green fluorescent protein (GFP) cDNA were incubated for 24 hours in either 50 ng/mL rIL-6 or vehicle control (1% BSA). 1 × 10^6^ cells were then injected into the tail vein of 4- to 5-week-old female NCR-Nu mice with 10 mice for the control group and 9 mice for the IL-6 treated group (Taconic). At 10 days, the mice were sacrificed and lungs were dissected. Formalin-fixed gross lung tissue was examined for the presence of GFP-expressing tumor foci. The number and size of metastatic foci per field of examination was quantified from 5 random sites for each mouse using at 4× magnification using NIH ImageJ software. Lung tissue was then paraffin embedded and sectioned (5 μM) at the Stony Brook University Research Histology Core Lab. Sections were stained with hematoxylin and eosin for analysis.

### Statistical analysis

Data are expressed as the mean ± S.E. of triplicates. Each experiment was repeated at least three times. Student's *t* test was used to assess differences (*p < 0.05; **p < 0.01; ***p < 0.001).

## SUPPLEMENTARY MATERIALS FIGURES AND TABLES



## Abbreviations

The abbreviations used are:

MMP-14: matrix metalloproteinase-14
IL-6: interleukin-6
