# Supplementary material for: Interleukin-6 increases matrix metalloproteinase-14 (MMP-14) levels via down-regulation of p53 to drive cancer progression
Source: Oncotarget. 2016 Aug 12;7(38):61107–20. doi: 10.18632/oncotarget.11243 (PMC5308639; doi:10.18632/oncotarget.11243)
Supplement: Supplementary file 1 [file oncotarget-07-61107-s001.pdf]

# Interleukin-6 increases matrix metalloproteinase-14 (MMP-14) levels via down-regulation of p53 to drive cancer progression

## Supplementary Materials

**Supplementary Table S1: Primers used for cloning, site-directed mutagenesis, and real time PCR**

| Primer Description | Forward (5′ > 3′) | Reverse (5′ > 3′) |
|---|---|---|
| Cloning of 1.2 Kb Promoter from HT-1080 Cells (A) | ATGGTACCAACATGGTGAAACCCTGTCC | ATCCCGGGTTTAACTCCAAGCCG ACAGC |
| Truncation B | ATGGTACCCTCCCACACTTTTCCTGC | ATCCCGGGTTTAACTCCAAGCCG ACAGC |
| Truncation C | ATGGTACCCAGATCCCACGGCCTTG | ATCCCGGGTTTAACTCCAAGCCG ACAGC |
| Truncation D | ATGGTACCGACCCCACCACCATCCCACAC | ATCCCGGGTTTAACTCCAAGCCG ACAGC |
| Deletion 1 to Truncation D (D1) | ACATAGCCCCCAATAATTC | GGTACCTATCGATAGAGAAAAG |
| D2 | AATACCAGAGGAATCAAGC | AGCATTTGTCTCACCTCAG |
| D3 | CCCTGGATCCCCCTACAG | CAGCATTTGTCTCACCTCAG |
| D4 | ATGGCAGCCTGCACCACA | GACGGGATGTGGGAGACTTTG |
| D5 | TTTTTTTCCTTCCAGTTCTTGGTTGTAATTGG | CTGCAGGGGTTGAGGCCG |
| D6 | ACGTCCCCAACCAGGAAA | AAAAAAACACCTCTAAGTTGCC |
| D7 | TGTGGGAGAAGGGAGGGA | GGTTGTTTTAGCCTGAATCCAATT AC |
| D8 | AGAGAGGGAACCAGACCCC | GCCTCTCCTCCGTCCCCG |
| Real Time Primers for HPRT-1 | ACCCCACGAAGTGTTGGATA | AAGCAGATGGCCACAGAACT |
| Real Time Primers for MMP-14 | GAGCTCAGGGCAGTGGATAG | GGTAGCCCGGTTCTACCTTC |
| Real Time Primers for p53 | ATCTTGGGCCTGTGTTATCTCC | TCCAGTGTGATGATGGTGAGG |

## Supplementary Table S2: Primers used for chromatin immunoprecipitation (ChIP)

| Primer Description | Forward (5′ > 3′) | Reverse (5′ > 3′) | Amplicon Length (bp) |
|---|---|---|---|
| Amplifying the MMP-14 promoter region in which p53 and Sp1 are predicted to bind | CCTTCCAGTTCTTGGTTGTAA TTGG | CTCCCACAGCCTCTCCTCC | 101 |
| Amplifying the MMP-14 promoter region in an area where p53 and Sp1 are not predicted to bind | ACCCTGAGGTGAGACAAATGC | GACGGGATGTGGGAGACTTTG | 86 |
| Amplifying p53 predicted binding site in p21 promoter | GAGGTCAGCTGCGTTAGAGG | CATCTCAGGCTGCTCAGAGT | 75 |

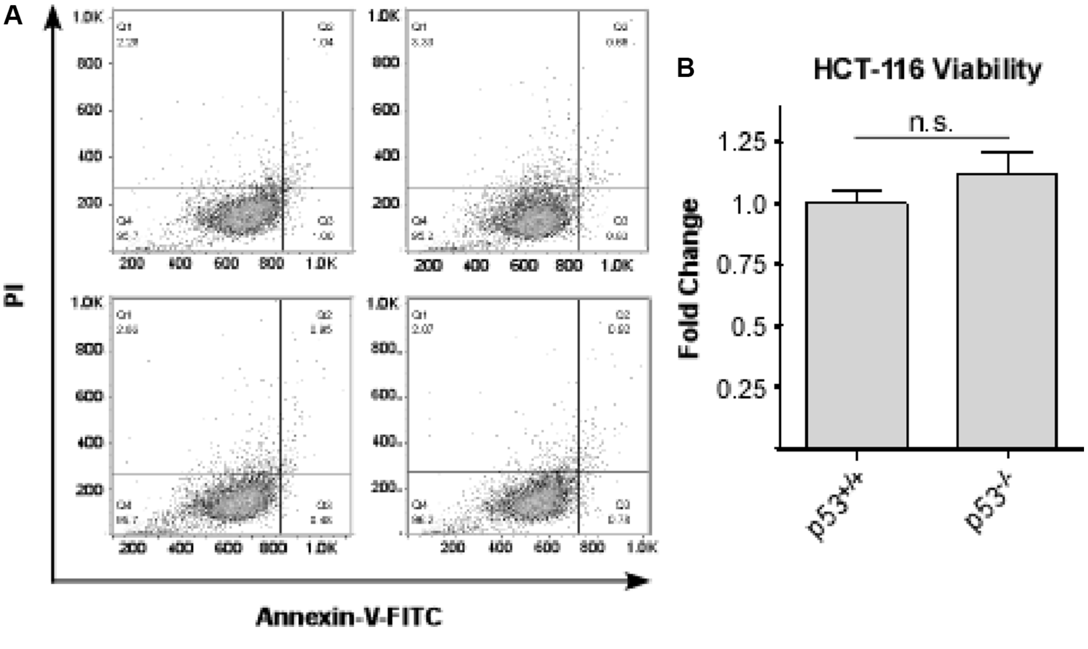


**Supplementary Figure S1: The observed phenotypic changes are not a result in differences in cell viability.** (**A**) HT-1080 cells were transiently transfected to overexpress p53 or vector control. After 24 or 48 hours post-transfection cells were stained with Annexin V-FITC and propidium iodide (PI) and subjected to flow cytometric analysis. No differences in apoptosis were observed.(**B**) Differences in viability of $p53^{+/+}$ and $p53^{-/-}$ HCT-116 cells was measured using MTT assay. No significant differences in cell viability were observed.

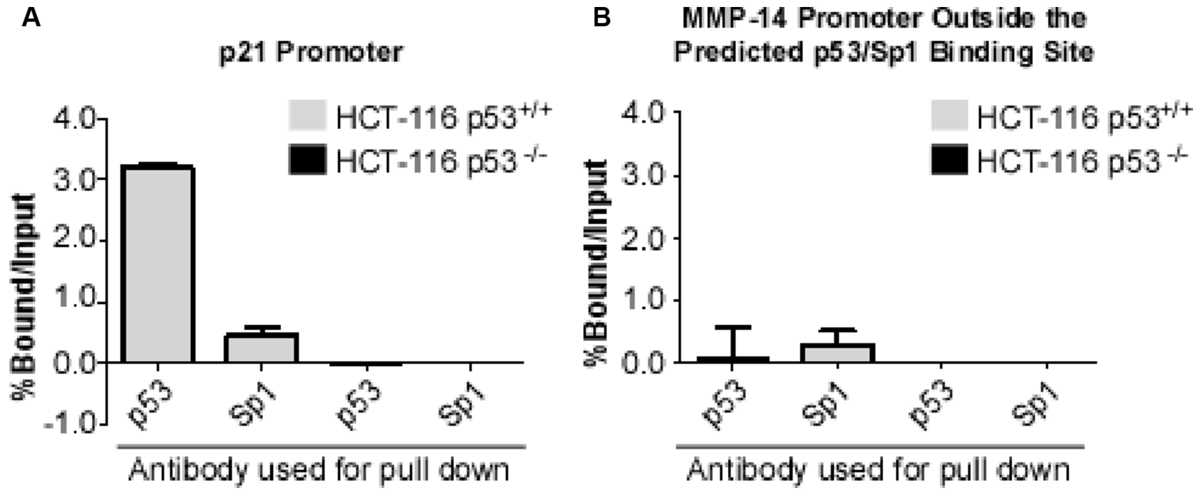


**Supplementary Figure S2: Validation of ChIP assay.** (**A**) p53 binds to the p21 promoter in HCT-116 WT cells but not in the p53 null cells. There is minimal binding of Sp1 to the p21 promoter in either strain. (**B**) There is minimal binding of p53 or Sp1 to the MMP-14 promoter in a region outside the predicted binding site.

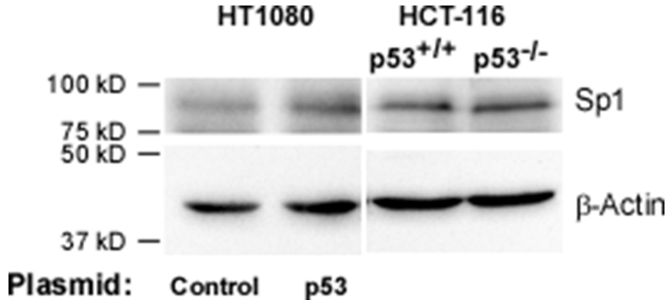


**Supplementary Figure S3: Sp1 levels are consistent in both HT-1080 and HCT-116 cells regardless of p53 status.**

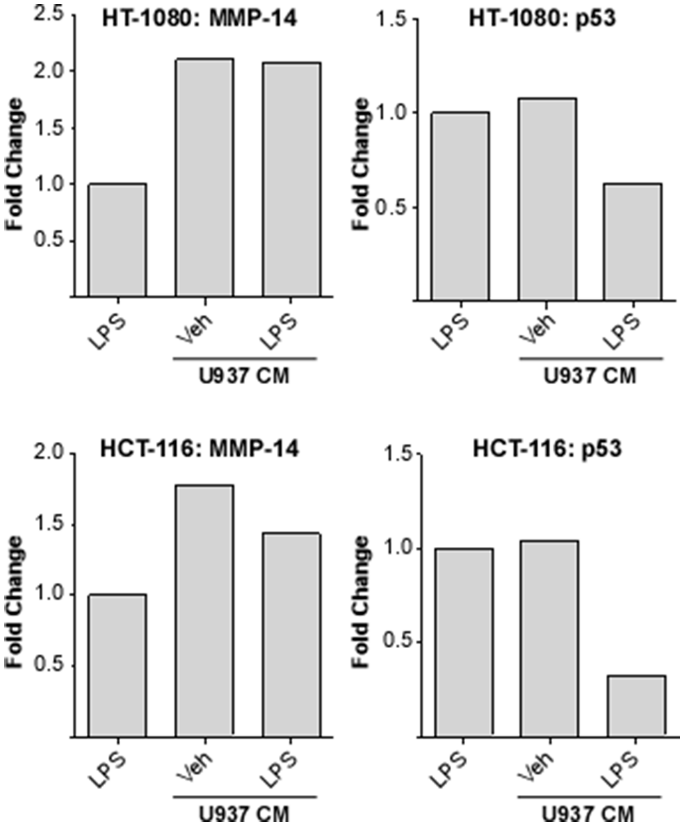


**Supplementary Figure S4: Densitometric analysis of MMP-14 and p53 levels in HT-1080 cells (top) and HCT-116 cells (bottom) treated with LPS alone or with conditioned media collected from U937 macrophage cells stimulated with LPS to induce pro-inflammatory cytokines or vehicle control.**

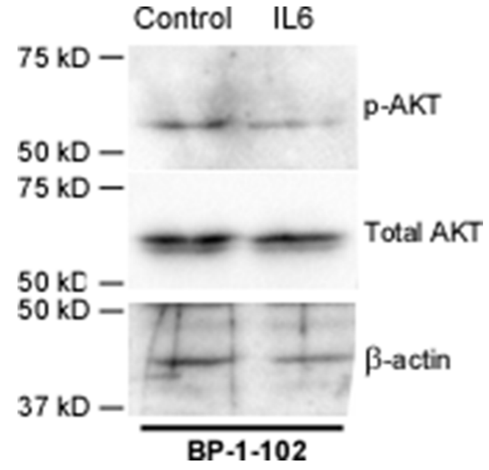


**Supplementary Figure S5: Inhibition of p-Stat3 in HCT-116 $^{+/+}$ cells using 10 ug/mL BP1-102 for one hour prior to 15 minutes of IL-6 stimulation abrogates phosphorylation of AKT.**
